# Mineralized collagen-modified PMMA cement enhances bone integration and reduces fibrous encapsulation in the treatment of lumbar degenerative disc disease

**DOI:** 10.1093/rb/rbz044

**Published:** 2019-12-02

**Authors:** Long Yang, Jianjun Kong, Zhiye Qiu, Tieliang Shang, Siyu Chen, Rui Zhao, Maria Grazia Raucci, Xiao Yang, Zhanyong Wu

**Affiliations:** 1 National Engineering Research Center for Biomaterials, Sichuan University, Chengdu 610064, China; 2 Department of Orthopaedics, Orthopaedic Hospital of Xingtai, Xingtai 054000, China; 3 Department of Orthopedic Laboratory, Xingtai Institute of Orthopaedics, Xingtai 054000, China; 4 Beijing Allgens Medical Science and Technology Co., Ltd, Beijing 102609, China; 5 School of Materials Science and Engineering, Tsinghua University, Beijing 100084, China; 6 Institute of Polymers, Composites and Biomaterials, National Research Council of Italy, Naples 80125, Italy

**Keywords:** mineralized collagen, lumbar degenerative disc disease, percutaneous cement discoplasty, fibrous encapsulation

## Abstract

As a minimally invasive surgery, percutaneous cement discoplasty (PCD) is now contemplated to treat lumbar disc degeneration disease in elder population. Here, we investigated whether the osteogenic mineralized collagen (MC) modified polymethylmethacrylate (PMMA) cement could be a suitable material in PCD surgery. Injectability, hydrophilicity and mechanical properties of the MC-modified PMMA (PMMA-MC) was characterized. The introduction of MC did not change the application and setting time of PMMA and was easy to be handled in minimally invasive operation. Hydrophilicity of PMMA-MC was greatly improved and its elastic modulus was tailored to complement mechanical performance of bone under dynamic stress. Then, PCD surgery in a goat model with induced disc degeneration was performed with implantation of PMMA-MC or PMMA. Three months after implantation, micro-computed tomography analysis revealed a 36.4% higher circumferential contact index between PMMA-MC and bone, as compared to PMMA alone. Histological staining confirmed that the surface of PMMA-MC was in direct contact with new bone, while the PMMA was covered by fibrous tissue. The observed gathering of macrophages around the implant was suspected to be the cause of fibrous encapsulation. Therefore, the interactions of PMMA and PMMA-MC with macrophages were investigated *in vitro.* We discovered that the addition of MC could hinder the proliferation and fusion of the macrophages. Moreover, expressions of fibroblast-stimulating growth factors, insulin-like growth factor, basic fibroblast growth factor and tumor necrosis factor-β were significantly down-regulated in the macrophages cocultured with PMMA-MC. Together, the promoted osteointegration and reduced fibrous tissue formation observed with PMMA-MC material makes it a promising candidate for PCD surgery.

## Introduction

Lumbar degenerative disc disease (DDD), characterized by intervertebral lumbar disc narrowing, reduced disc height, and loss of disc signal intensity, is a condition associated with the degeneration of one or more of the discs in the spine [1, 2]. The radiographic findings of DDD are discovered in 40% of individuals younger than 30 and in more than 90% of individuals older than 50 years of age [3]. To treat symptomatic DDD, strategies always start with non-surgical therapies, such as activity modification, medications and physical therapy [4]. After several months of these conservative approaches, patients who fail to improve their condition, surgical operations such as disc replacement and spine fusion shall be considered [5]. However, standard surgical treatment for DDD of elderly patients is often restricted. In the ‘aging spine’ population where, besides disc degeneration, osteoporosis is the leading pathology, the vertebral endplate often loses its integrity and shows variety forms of fragmentation. Applying commercially pre-shaped interbody spacers in these discs generates a higher risk of postoperative segmental collapse and cage subsidence [6]. Minimally invasive surgeries are more suitable in elder population, since they reduce the risk of complication and thus the surgical morbidity. As such a minimally invasive technique, percutaneous cement discoplasty (PCD) was first developed by Varga *et al* [7]. who used polymethylmethacrylate (PMMA) material as an intervertebral spacer in 1995. In order to restore segmental height, PMMA bone cement was later implanted into the intervertebral disc space, as part of further development [8]. The primary advantage of using PMMA is that it can be shaped individually, thereby adapting its load-bearing surface perfectly to the shape of the patients’ endplates. Furthermore, the viscous form of PMMA can fill up the space between fragmented endplates and provide a stabilizing effect when it solidified. Varga *et al*. [6] demonstrated that after PCD surgery with KyphX^®^ bone cement, majority of the DDD elder patients reported a significant reduction in the lower back pain postoperatively. At 6-month follow-up, more than 50% of the patients had a minimum 10-point decrease in their Oswestry disability index scores. In 2016, Yamada *et al*. [9] found that the 109 elderly patients with degenerative lumbar scoliosis had sustained clinical benefit for at least 2 years after percutaneous intervertebral PMMA bone cement injection. More recently, Sola *et al*. [10] reported that a 78-year-old woman who had degenerative scoliosis with multiple pneumodisk was soon able to stand up and walk well after PCD surgery.

These studies demonstrated a promising strategy of adopting PCD surgery to treat symptomatic DDD of elder population. However, PMMA cement used in these studies is a bioinert material which form no chemical or biological bonding with host bone at the implant surface, resulting in always being covered by fibrous tissue without osteointegration at the implant site [11, 12]. The layer of fibrous tissue in between cement and bone is recognized as the weak-link zone and often lead to loosening of the implanted PMMA at the cement-bone interface [7]. In addition, the compressive elastic modulus of normal human vertebral body is several folds higher than that of the natural human vertebral body [11]. Several approaches have been employed to improve PMMA cement-bone interaction [7, 13, 14]. One is achieved by incorporation different types of bioceramics into PMMA during surgical preparation process, such as hydroxyapatite (HA), β-tricalcium phosphate or silicon-based bioglass. These bioactive fillers could facilitate the mineralization process biomimetically and prevent the formation of unfavorable fibrous capsule. Among these additive materials, mineralized collagen (MC) stands out as a biomimetic material with the same chemical composition and hierarchical structure to the natural bone. Researches and clinical practices showed that the MC is able to enhance new bone regeneration at various bone defect sites [11]. Yu *et al*. showed an effective result of MC in anterior cervical interbody fusion. A total of 91 patients with symptomatic cervical disc diseases were allocated for either autologous bone graft or MC randomly. In their study, MC was demonstrated to be a promising substitute for autologous bone graft, in terms of maintenance of lordotic angle, vertebral height and rate of fusion [15]. Furthermore, Wang *et al*. [16] and Zhu *et al*. [17] had compared the clinical effect of MC incorporated PMMA and pure PMMA for the treatment of vertebral compression fractures in osteoporotic patients. Excellent compatibility with adjacent tissue was achieved in the MC-modified PMMA group of patients. There was a noticeable increase in surrounding trabeculae than the pure PMMA group. Moreover, MC incorporated bone cement significantly lowered the incidence of postoperative adjacent vertebral fracture by approximately 10%, and increased bone mineral density of the treated vertebral bodies [16].

To summarize, MC alone was demonstrated to be an osteogenic biomaterial for bone regeneration including intervertebral fusion, and MC-modified PMMA was clinically used to treat vertebral compression fractures. However, whether MC-modified PMMA could provide an added benefit in the intervertebral PCD surgery is still unknown. Thus, the current study aimed to assess the application of MC-modified PMMA in PCD of a goat model, with minimally invasive operation. First, injectability, stability, hydrophilicity and mechanical properties of the composite material were documented. After 3 months of implantation, bone integration and fibrous tissue formation around the material were characterized by micro-computed tomography (CT) and histological analysis. Finally, a possible cellular to molecular level mechanism was proposed to facilitate understanding of the *in vivo* results.

## Materials and methods

### Preparation of MC particles

**Figure 1:**
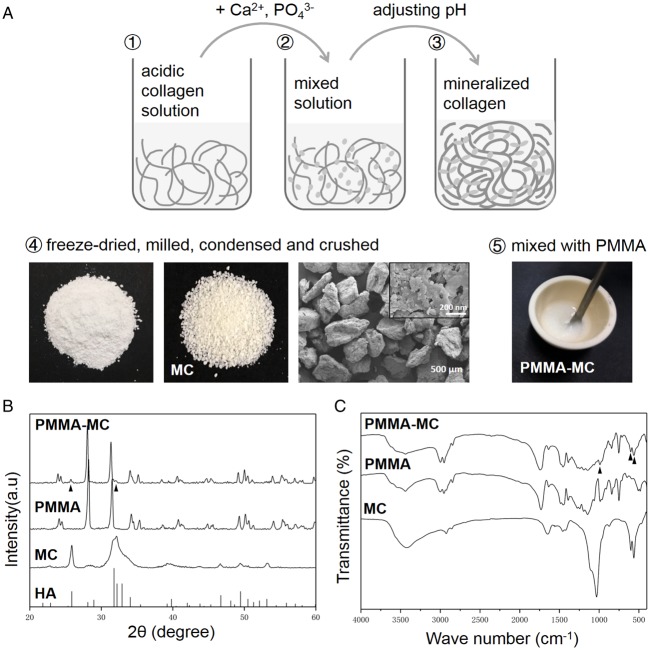
(**A**) Preparation of the MC particles and modified PMMA. (**B**) XRD of the MC, PMMA and PMMA-MC materials and hydroxyapatite standard card. (**C**) FTIR spectra of the MC, PMMA and PMMA-MC materials. All samples wer analysed in powdered form

The preparation of MC particles was performed according to the previous reports [18, 19]. As shown in Fig. 1A, water-soluble phosphate salt and calcium salt solution were dropped into acidic type I collagen solution. Sodium hydroxide solution was used to adjust pH of the reaction system to 7.4. Then the deposition of MC was gradually formed. In this process, the nucleation and growth of HA crystals were guided by collagen macromolecular template, which was similar to the mineralization process of the natural bony tissue [18, 19]. After reacting for 48 h, the resultant deposition was collected, purified by centrifugation with deionized water for several times. After freeze-drying and milling, the product was passed through a 200-mesh stainless steel screen to obtain MC powder. The powder was subsequently placed in a customized mold and compressed into a compact form with 1000 MPa pressure maintained for 40 s. Each compressive pressure was maintained for at least 30 s. The densified MC was thus fabricated. Next, this bulk material was mechanically crushed into particles. To deliver PMMA cement in minimal invasive surgeries of spine, the inner diameter of PKP or PVP device is usually 2.5–4.0 mm, thus MC particles of 300–400 μm in size were used in this study according to the previous work [11].

### Preparation of PMMA-MC bone cement

In the modification process, powder and liquid (2.6: 1, w/v) parts of the PMMA bone cement (Osteopal^®^V, Heraeus Medical GmbH, Germany) were first mixed. Then, a uniform flowing phase was obtained by manual mixing the liquid and the powder components with a spatula in a teflon container for 30 s. According to our previous work, 15 wt% MC particles were added into mixture [11]. The mixture was filled into teflon molds with dimensions of 20 mm in length, 10 mm in width and 2 mm in thickness to obtain rectangular samples for tests. The MC-modified PMMA bone cement group used in the current study were thus obtained (PMMA-MC). Besides, the unmodified commercial bone cement was considered as a control group (PMMA). The processing time and maximum temperature of these two cements were measured during the preparation process mentioned above. The processing time of a bone cement consisted of four standard phases, including mixing time, waiting time, application time and setting time. Among them, application time and setting time are the most important. The former indicates the time from the bone cement being applicable to being difficult to inject, and the latter indicates the time from injection of the bone cement to it becomes hardened. The retention in polystyrene sponge and solidification in PBS at 37°C of the two bone cements were also recorded.

### Materials characterization

#### Scanning electron microscopy and energy dispersive spectrometry (SEM/EDS)

Morphology of the sample surface was investigated by a field-emission scanning electron microscopy (FE-SEM, S4800, Hitachi, Japan) assisted with an energy dispersive spectrometry (EDS). The MC particle samples were dried in a desiccator for 24 h before the analysis. The samples were then mounted on aluminum stubs using conductive carbon tape, and then coated with a gold layer. Subsequently, morphology of the samples was examined at an accelerating voltage of 5 kV with different magnifications. Moreover, the implanted sample embedded in epoxy as described in 2.6.4 were also studied with SEM to evaluate the interface of the cement and new bone matrix. Morphologies of implanted sample were examined at an accelerating voltage of 20 kV and working distance of 10 mm. The SEM rendered images were pseudo-color processed by using a Matlab routine to distinguish different components by color.

#### X-ray diffraction

Phase composition of the bone cement samples was investigated by X-ray diffraction (XRD, AXIS Ultra DLD, Kratos, UK). The samples of PMMA, MC and PMMA-MC were pulverized, passed through a 200-mesh sieve and the fine powder was placed in a 37°C constant temperature oven to a constant weight. The diffraction was performed using a Cu Kα radiation source with a wavelength of 1.5405 Å at a voltage of 40 kV and a current of 35 mA. The results were compared to a standard card for HA (International Centre for Diffraction Data (ICDD) PDF card #9-432) and the difference in phase composition was observed. The patterns were analysed with MDI Jade software (v.6.5, Materials Data, CA, USA) with PDF cards (PDF2-2004, ICDD, Newton Square, PA, USA).

#### Fourier transform infrared spectroscopy

Fourier transform infrared spectroscopy (FTIR, Nicolet 6700, Thermo, USA) was used to characterize the molecular structure of the samples. The samples of PMMA, MC and PMMA-MC were prepared similar to those in the XRD analysis. Before the FTIR test, 10 mg of the samples were mixed with 1 g potassium bromide (KBr), and the mixture was transferred to a mortar and grind to a fine powder in an agate mortar incubated with infrared light. Next, a small amount of fine powder was compressed into a thin transparent pellet using a hydraulic press. Analyses were performed in the range 400–4000 cm^−1^ with a resolution of 4 cm^−1^. The samples were scanned 64 times for each FTIR measurement and the spectrum acquired was the average of all these scans. The data of the experiment were processed and analysed by OMNIC 8.0 infrared software (Thermo Fisher Scientific Inc. Madison, WI, USA).

### Wetting property of the bone cement

Wettability of the samples was evaluated by a water contact angle measurement machine (lL4200, KRÜSS GmbH, Germany). The wettability of a surface is defined as the contact angle (θ) formed between the solid surface and the tangent drawn at the liquid drop [20]. Before the experiments, the PMMA and PMMA-MC samples were sanded using 400, 800 and 1200 cc sandpaper and polished to obtain a flat surface. Then, the samples were sonicated in an ultrasonic cleaner bath for 30 min and dried for 24 hours. A droplet (4 μl) of deionized water was deposited on the surface of the samples at room temperature. The software provided with the instrument allows automated measurements of the static contact angle. The values are obtained using Laplace–Young curve fitting and expressed in degrees. At least five measurements were carried out on different areas of each sample.

### Dynamic mechanical property of the material

The viscoelastic properties of a material, including the ability to dissipate mechanical energy (damping) and the ability to recover from deformation (elasticity), can be measured by applying an oscillatory stress to a specimen and measuring the resultant oscillatory strain. This type of mechanical testing is usually performed with a dynamic mechanical analyzer (DMA) as described in our previous works [21, 22]. Before the experiments, the rectangular samples of PMMA and PMMA-MC were obtained from teflon molds with dimensions as previously described. The samples were mounted on a DMA device (DMA Q800, TA Instruments, New Castle, DE, USA) with a three-point bending configuration (span length = 15 mm). The test was conducted under displacement control. Scanning frequencies ranged from 0.1 Hz to 10 Hz. The samples were measured at amplitude of 20 μm with 10 mN pre-stress. Three parallel samples were performed, and the average values of storage modulus (*E*’) and tan *δ* were obtained*. E′* is equivalent to Young's modulus in static test and tan *δ* is an indicator of the amount of energy dissipated by viscous mechanisms relative to energy stored in the elastic component.

### *In vivo* animal study

#### Experimental animals and groups

The animal experiments were approved by the Animal Care and Use Committee of Orthopaedic Hospital of Xingtai. A total number of eight goats were purchased and housed in the animal experiment center of Hebei Medical University (18 months old, 26–33 kg). The goats were first housed and acclimatized for 7 days in animal breeding room prior to the surgery. They were randomly divided into three groups for three different material treatments: Titanium cage implant (Ti-cage group, two goats), MC-modified PMMA implant (PMMA-MC group, three goats) and PMMA implant (PMMA group, three goats). Titanium cage is used as a negative control in this study. It is a bioinert implant material which is often used in clinical bone fusion, especially in spine surgeries [23, 24]. It is also widely used as a control group to be compared with those bioactive implants in assessment of their bone fusion efficiency [25, 26]. Each animal was operated with L3–L4 lumbar vertebral space. All surgeries were carried out at the Hebei Medical University.

#### Surgical procedure

The goats were fasted strictly 24 h for food and 12 h for water prior to surgery. The animals were weighed at the time of implantation. The goats were sedated by injection xylazine hydrochloride (0.05 mg/kg body weight intramuscular) and local infiltration of 1 ml 2% lignocaine with adrenaline (1:200 000). The skin from the left flank and mid back was shaved and disinfected. After that, the goats were positioned in left lateral recumbence on the operating table. A lateral retroperitoneal approach was used to expose the lateral aspects of L3–L4. The nucleus pulposus tissues of the three-consecutive lumbar IVDs were aspirated to induce disc degeneration. A volume of 1.0 ml PMMA cement or PMMA-MC cement was injected into each hole using an injection gun, making sure that the implants were at proper positions. Commercialized titanium cage was implanted at the same area of two goats which were served as positive controls. The surgical wound was closed in layers. The goats were left to fully recover from the anesthesia in the room and returned to their individual cages. Close observation was kept every day. Penicillin and painkiller were intramuscularly delivered for 3 days postoperatively. Animals were euthanized after 3 months. Prior to the further analysis, the harvested lumbar spine bone including the implant and the surrounding host bone tissue were immediately fixed with 4% paraformaldehyde solution for 7 days.

#### Micro-CT analysis

High-resolution micro-CT images were used to assess new bone formation surrounded the implanted materials. The scanning was performed with X-ray tube set at 70 kVp and 114 µA (µCT80, Scanco Medical, Bassersdorf, Switzerland). Integration time (300 ms) was required for each sample and the region of interest had a resolution of 30 µm. The generated grey scale images were then reconstructed and analysed with the Scanco software. According to our previous work, a global threshold was adopted to differentiate the materials from bony tissue [27–29]. Three-dimensional (3D) images were extracted from the operated intervertebral lumbar region to visualize the connectivity between the implants and the new bone. Implant circumference and the length of all circumferential segments with direct contact between implant and bone were measured. Circumferential contact index (%) of the PMMA and PMMA-MC groups was calculated in accordance with literature [30].

#### Histological observation

As mentioned previously, the material-implanted goat vertebral bone tissues were harvested and fixed in 4% paraformaldehyde for 7 days. The specimens were dehydrated in ascending concentrations of alcohols from 75% to 100% and then embedded in epoxy [31]. A total of 15-μm sections were obtained with hard tissue slicer (Leica Cryocut, Germany). The sections were stained by methylene blue and basic fuchsin for histological observation. Then, the histological overview of each slide was observed under light microscope to assess the bone-cement integration by a light microscope (Olympus Bx60) equipped with a digital CCD camera.

### *In vitro* macrophage co-culture

Mouse RAW 264.7 macrophages purchased from Cell Bank of Chinese Academy of Sciences (Shanghai, China) were used in this work. After PMMA and PMMA-MC discs were placed into the culture plate, a density of 1 × 10^5^ cells per well were seeded. The culture medium used was DMEM (Gibco, USA) supplemented with 10% fetal bovine serum (Gibco, USA) and 1% penicillin/streptomycin (Gibco, USA). The survival status of macrophages cultured on different material groups was stained with FDA/PI and observed by a confocal laser scanning microscopy (CLSM, Leica TCS-SP5, Germany). MTT assay was performed to quantify cell proliferation at days 1, 4 and 7 in accordance with our previous macrophage culture study [32]. For morphological observation and quantification of cell fusion event, actin skeleton of the cells was labeled with Phalloidin-TRITC (Sigma, USA) for 30 min. After rinsing with PBS, the cell nuclei was contrast-labeled in blue by 4′,6-diamidino-2-phenylin dole dihydrochloride (DAPI, Sigma, USA) [33].

To investigate the effect of MC on inflammatory and fusion-related gene expressions, cell culture of consecutive days was collected for RNA isolation and quantification. The samples were gently rinsed three times with PBS, and an RNeasy Mini Kit (Qiagen, Germany) was used to extract the total RNA. Then, to reverse-transcribe the total RNA into complementary DNA (cDNA), an iScript^TM^ cDNA Synthesis Kit (Bio-Rad, CA, USA) was used with 20-μl reaction system. With SoFast^TM^ EvaGreen^®^ Supermix (Bio-Rad, USA), real-time quantitative PCR reaction was conducted (CFX960, Bio-Rad, USA). The amplification procedure was an incubation at 95°C for 2 min, followed by 40 cycles of 95°C for 2 s and finally 60°C for 5 s. The relative fold change was computed using the ΔΔCt method. The gene expression levels of interleukin-1β (IL-1β), IL-6, tumor necrosis factor-α (TNF-α), macrophage chemotactic protein 1 (MCP-1), macrophage inflammatory protein (MIP), insulin-like growth factor-1 (IGF-1), basic fibroblast growth factor-1 (bFGF), tumor necrosis factor-β (TGF-β), vascular endothelial growth factor (VEGF), IL-4 and IL-13 were measured. The GAPDH was selected as the housekeeping gene to normalize the expression levels of target genes. To further confirm the gene expression result at the protein level, Western blotting analysis of the proteins with regard to differentially expressed genes was conducted according to our previous method [33]. The primary antibodies to TNF-α, IGF-1, bFGF, TGF-β and IL-4 were purchased from Cell Signalling, MA, USA. Protein bands were visualized using a ChemiDocTM XRS+ image system with image LabTM software (Bio-Rad, USA).

### Statistics analysis

All data were collected from at least three samples for each experiment and expressed as the mean ± standard deviation. Statistical analysis of the data was performed using Student’s *t* test with a level of significance of *P* < 0.05.

## Results and discussion

Lumbar DDD is a multifactorial process with changes in disc architecture and integrity, which can cause severe chronic pain in the lower back which can radiate to hips and legs [2]. It is one of the main causes of disability worldwide, imposing enormous socio-economic burden and clinical costs to society [34]. In the general population, the prevalence of radiological signs of lumbar DDD increases with increasing age [35]. Consequently, physicians will face an ever-increasing number of elderly patients suffering from DDD in the future [36]. Minimally invasive surgery is one of the most preferred approaches to treat elder patients with lumbar disease, due to the decrease in surgical time, intraoperative blood loss, infection rates, hospital length of stay and cost [10]. Thus, in the treatment of DDD, percutaneous cement discoplasty is contemplated worldwide. As mentioned previously, Varga *et al*. [6] demonstrated that after the PCD surgery with PMMA bone cement, majority of the DDD patients had a significant reduction in their lower back and leg pain postoperatively. He further concluded that elderly patients with symptomatic dynamic foraminal stenosis and vacuum phenomenon in the intervertebral disc are suitable candidates for PCD, particularly if they represent high-risk patients for open surgery. In the present study, we investigated whether MC-modified PMMA bone cement could be adopted in the PCD surgery with a goat model. Bone integration and fibrous tissue formation around the implant were characterized in the PMMA-MC group, comparing with the traditional PMMA and Ti-mesh control groups.

Surface morphology of the fabricated MC particles was demonstrated in Fig. 1A. As can be observed from the SEM image, the average size of the MC particles used in the current study was about 300–400 μm. Moreover, the MC particles exhibited an irregular shape. The higher magnification image presented MC fibers. There are nanoscale particles adhered on the surface of the fiber. Based on the result shown in Fig. 1B, the diffraction peaks of MC widened and matched well with the standard reference of HA, indicating that the mineral composition formed during the biomimetic mineralization was poorly crystallized HA. In order to detect whether there is any MC in PMMA-MC, the phase composition of PMMA and PMMA-MC was identified by XRD. The presence of the PMMA peaks was clearly observed in the pattern of the PMMA-MC group. Moreover, Specimens of PMMA-MC revealed diffraction peaks at 2θ =  25.74° and 31.65°, which is consistent with the diffraction peaks of MC. Thus, according to their diffraction peaks, PMMA-MC were composed of PMMA phase and MC phase. The FTIR spectra of MC, PMMA and PMMA-MC are shown in Fig. 1C. The MC presented characteristic peaks of type-I collagen specifically at 1653 cm^−1^ (amide I) and 1030 cm^−1^ (C-OH). Another two bands at 600 and 563 cm^−1^ were the phosphate group (${\text{PO}}_{4}^{3}$^−^). The spectra of PMMA and PMMA-MC presented the similar characteristic bands, including CH stretching vibration at 2952 cm^−1^, C=O stretching vibration at 1743 cm^−1^, CH3 stretching vibration at 1454 cm^−1^, as well as –O–CH3 stretching vibrations at 1145 cm^−1^. Moreover, the characteristic bands of MC occurred on PMMA-MC too (as indicated by arrow head). These results confirmed that the prepared PMMA-MC contained both MC and PMMA components.

**Figure 2:**
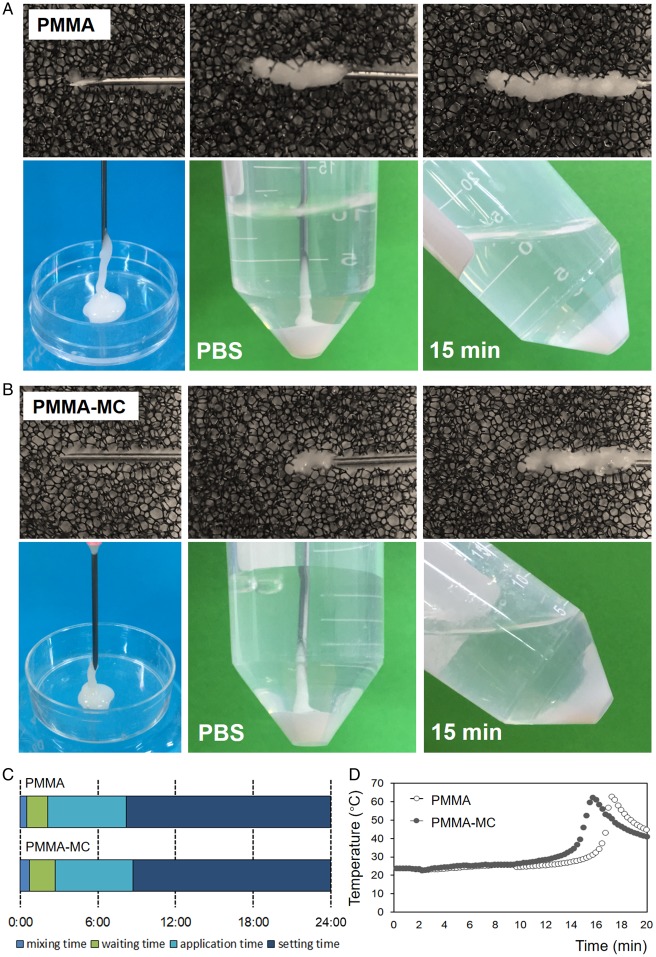
The retention in polystyrene sponge and solidification in PBS at 37°C of the PMMA (**A**) and PMMA-MC cements (**B**); comparison of the processing time between two cements (**C**); characterization of the maximum temperature of two cements (**D**)

**Figure 3:**
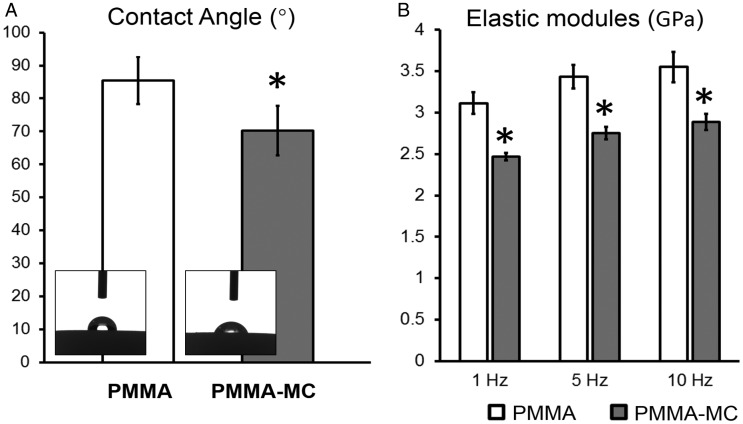
Water contact angle (**A**) and dynamic elastic modules at different strain frequencies (**B**) of the PMMA and PMMA-MC cements, *P < 0.05 denotes for significant difference as compared to the PMMA group

Processing time and injectability of the bone cement are important operation parameters when performed by an orthopaedic surgeon. Osteopal^®^V, the PMMA bone cement used in the current study, has an application time of about 6 minutes, and a setting time of about 16 minutes under the conditions of 23°C and 50% relative humidity. The addition of MC granules did not show significant variation on the time of these two phases (Fig. 2A–C), which makes no change to the operation habits of surgeons. During the application time, the hands-on experience to inject the PMMA-MC cement is similar to that of the pure PMMA cement in a blind test. The injected cement, whether PMMA-MC or PMMA, could be well retained in either polystyrene sponge or PBS at 37°C. During the 15-minute setting time, the solidifying PMMA-MC and PMMA cement maintained their initial shape and no disintegration or dissolution was observed. Both cements had a similar maximum temperature of 64°C (Fig. 2D). Furthermore, the hydrophilicity of the solidified PMMA and PMMA-MC was evaluated by the static sessile drop method and the results are presented in Fig. 3A. According to the images, both PMMA and PMMA-MC were hydrophobic. However, the hydrophilicity of PMMA-MC is improved after the introduction of MC particles. Based on the measured data, the contact angle of PMMA-MC was significantly lower than that of PMMA. The surface wettability of biomaterials determines the biological cascade of events at the biomaterial/host interface [37]. An enhanced surface hydrophilicity was proven to be favorable for cell adhesion and spreading which can accelerate the osteointegration of an implant at an early stage [38, 39].

The elastic modulus of human vertebral body is within 500–2500 MPa, whilst the hardened PMMA bone cement has an elastic modulus of 3000–3700 MPa [11, 40]. The discrepancy between the mechanical properties of PMMA and vertebral bone is supposed to result in higher fracture risk for the adjacent vertebral bodies after augmentation [41]. Different strategies have been employed to decrease elastic modulus of the PMMA bone cement. One of the strategies attempted is to offer porosity in PMMA with addition of alginate, carboxymethylcellulose and gelatin microbeads [42]. The porous PMMA can promote the ingrowth of bony tissue into the material body, therefore providing more anchorage of the PMMA within the host bone. However, mechanical strength of the porous PMMA was too low to be applied clinically. In the current study, we focused more on dynamic elastic modulus of the MC-modified PMMA, since human body is experiencing load with a wide range of frequency arising from daily life activities [43]. The results of the three-point bending mode in DMA test in terms of dynamic elastic modulus are shown in Fig. 3B. The elastic modules showed a rising trend in both groups with frequency increased. Mean values of elastic modules of different groups at frequencies 1, 5, 10 Hz were analysed with the independent samples *t* tests. At three frequencies analysed, the specimens from the PMMA-MC group all had significantly lower elastic modules, 2.4–2.8 GPa, than that of the PMMA group (*P* < 0.01). This value is in accordance with a literature study which replaced up to 15 wt% polycrystalline HA in the powder component of the PMMA cement and found the elastic modulus of the harden material was reduced to 2–2.5 GPa [44]. A decreased elastic modulus of the MC-modified PMMA is due to the discrepancy of mechanical property between these two materials. The strength of MC is much lower than that of the PMMA, which can be approximately regarded as a pore-space structure [45]. In this study, MC was homogeneously distributed within the PMMA which disrupts the integrality of the cement, resulting in a decrease in overall mechanical strength.

**Figure 4:**
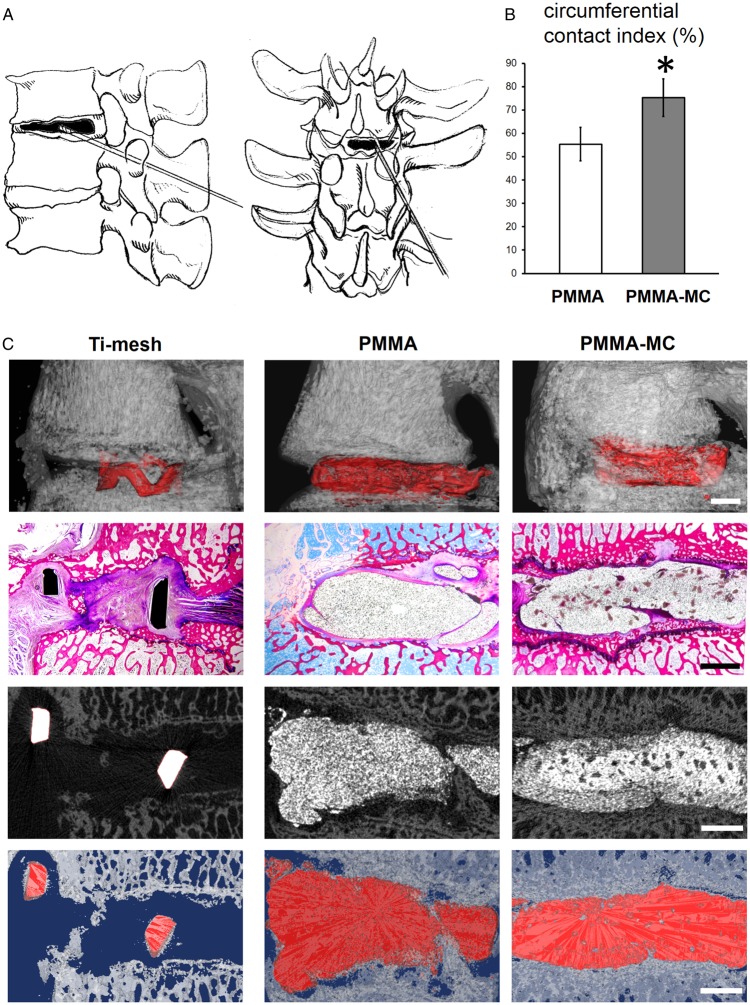
Gross observation of osteointegration. (**A**) Schematic diagram of goat L3–L4 vertebral space shows the site of minimally invasive PCD surgery. (**B**) Circumferential contact index (%) of the two cements with bone, *P < 0.05 denotes for significant difference as compared to the PMMA group. (**C**) General histological observation and micro-CT rendered image presents the intervertebral bone integration of the PMMA and PMMA-MC cements, scale bar: 2 mm

In the current study, the incorporation of MC granules was not only expected to grant the material a suitable elastic modulus but also designed to improve the biological activity of bone cement that will eventually enhance osteointegration of the implant. An enhanced implant osteointegration, represented by a large contact area of the interface, could make the load transfer more uniform and thus reduce the loosening rate. Therefore, 3 months post surgery, the bone integration and fibrous tissue formation around the bone cement were characterized. Circumferential contact index quantified from micro-CT reconstructed image revealed a significant higher value in the PMMA-MC group, meaning more direct contact between implant and bone (Fig. 4). The Ti-mesh control group showed no new bone formation in the defected area between vertebral bodies. A few gap area and discontinuity can be observed between implant and bone in the PMMA group. This radiolucent area is often regarded as fibrous tissue formation without mineral deposition [46]. Fibrous encapsulation would prevent direct interaction between bone and the implants. On the contrary, PMMA-MC group showed a continuous interface between implant and host bone. The radiographs rendered in this study were similar to a study conducted by Li *et al*. [47], who found a sharp and clear boundary between PMMA and defected rabbit femur bone, whilst a blurred interface with bone creeping substitution in the MC-modified PMMA implanted group.

**Figure 5:**
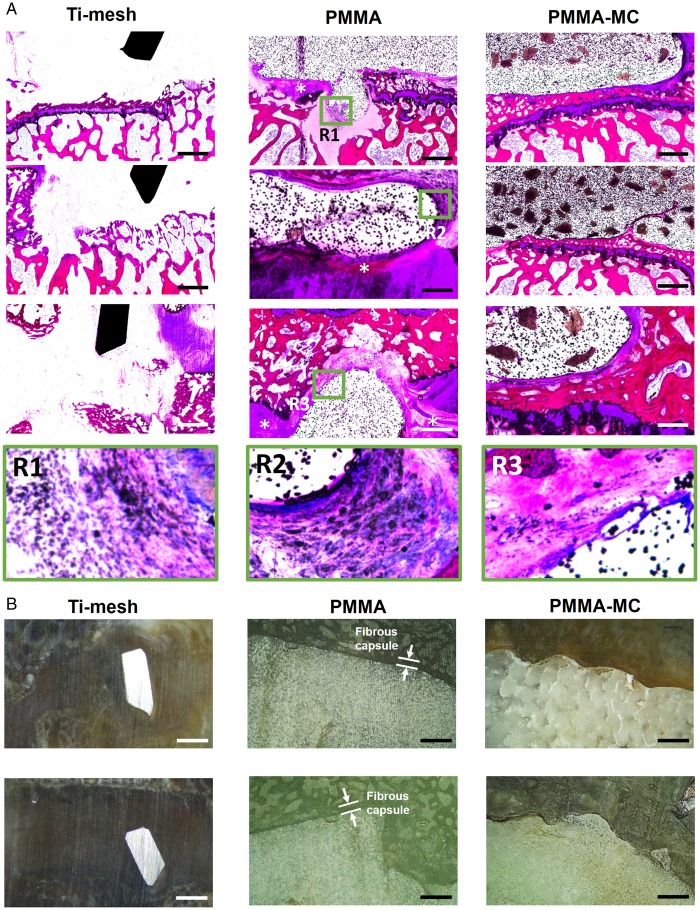
Osteointegration at the interface. (**A**) Histological observation of the interface between bone and different implants in a goat model, 3 months post surgery, scale bar: 1 mm. (**B**) Microscopic observation at the cross-sectional view of the embedded samples. *Gathering of macrophages, scale bar: 500 μm

**Figure 6:**
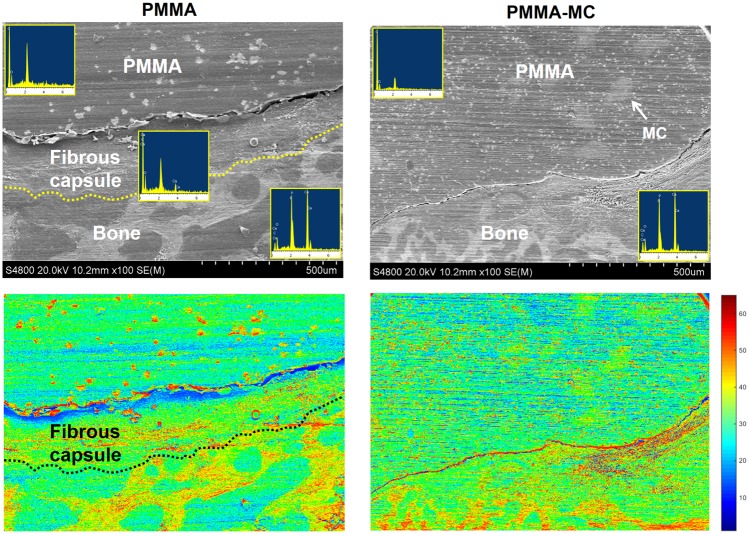
SEM/EDS Image shows the boundary between PMMA, PMMA-MC and host trabecular bone as well as the elemental analysis at respective regions

The osteointegration and fibrous tissue encapsulation around the implants were further visualized by enlarged histological staining images (Fig. 5A). The radiolucent area shown in the interface between PMMA and host bone was stained in purple color and presenting fibrous tissue feature. Higher magnification of the fibrous capsule demonstrated the presence of macrophages with high cell density (Fig. 5A, R1-R3). In contrast, new bone tissue was presented in the interface between PMMA-MC and host bone, as stained in dark red. Microscopic observation at the cross-sectional view of the embedded samples indicated an approximately 250 μm encapsulation layer surround the PMMA, which could impede mechanical performance of the implant (Fig. 5B). The bone bridging effect of the incorporated MC was demonstrated. Similarly, Sun *et al*. investigated the feasibility of using MC-modified porous sponge plug to preserve alveolar socket after tooth extraction. Histological analysis showed that new bone in the MC incorporated plug implanted dog group achieved synostosis: the boundary between the buccal and lingual bone plate was indistinguishable at week 12 [48]. SEM/EDS analysis (Fig. 6) further verified the boundary between PMMA and host trabecular bone were covered by fibrous tissue, with a distinct atomic ratio of C:O:Ca of 60:25:1 approximately [49]. PMMA-MC exhibited close contact with the surrounding bone tissue without interposed connective tissue. Moreover, this phenomenon can also be clearly observed on the respective pseudo-colored images.

**Figure 7:**
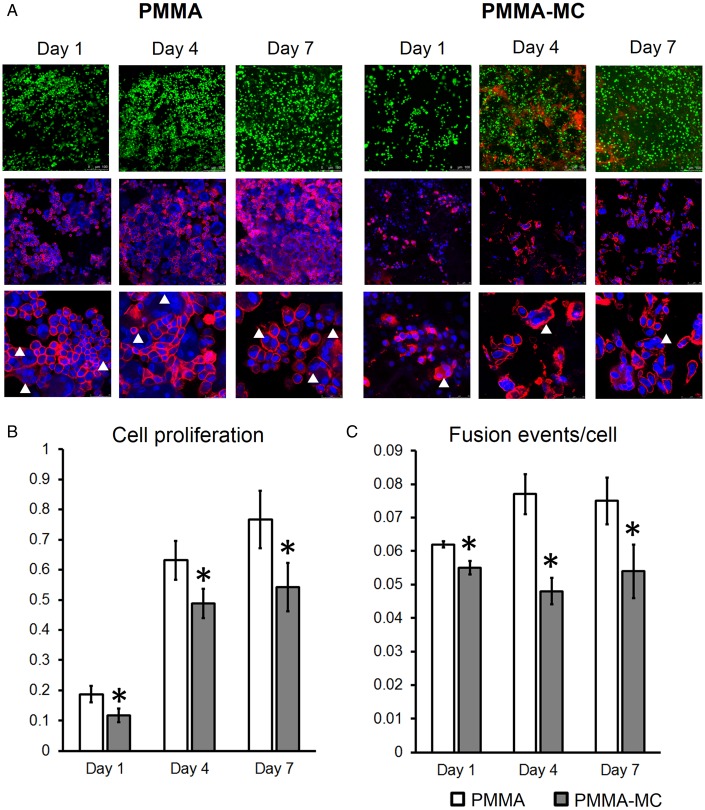
CLSM observation of live/dead cells (green/red), nuclei/actin skeleton (blue/red) (**A**), proliferation (**B**) and fusion events (**C**) of the macrophages cocultured with PMMA or PMMA-MC materials at days 1, 4 and 7, *P < 0.05 denotes for significant difference as compared to the PMMA group

In general, monocytes/macrophages are often activated at implant surfaces and modulate local fibroblast function, contributing to excessive deposition of collagenous matrix around implanted materials [50]. Thus, fibrosis and encapsulation of the biomaterials always correlated with a great number of macrophages gathering at implant site [51]. Moreover, it was reported that the fibrous capsule thickness is positively associated with macrophage density [52]. Literature studies demonstrated that macrophage fusion observed around implants and the growth factors secreted by activated macrophage are two critical factors that regulate fibro-proliferation [50]. Thus, in the current study, *in vitro* experiments were conducted using macrophages co-cultured on the surface of the solidified PMMA and PMMA-MC cements to unveil the underlying mechanism. A reduced macrophage cell proliferation was observed in the PMMA-MC group at days 1, 4 and 7 consistently (Fig. 7). Dead cells, as stained in red, were presented more on the surface of PMMA-MC, as compared to the PMMA. This result is consistent with a study conducted by Bhardwaj *et al*. who revealed an interesting phenomenon that introducing a nanophase HA coating on the titanium surface would significantly reduce bacteria and macrophage density. Similar to our study, their macrophage cell proliferation was inhibited at all times during the experiment, with incorporation of nanophase HA [53]. The interactions between cells were also presented to be weaker, in terms of a decreased cell fusion events observed in the PMMA-MC group. Cell fusion of macrophages gives rise to giant cell formation and contributed to collagenous encapsulation [54]. The incorporation of MC to PMMA can largely regulate cell proliferation and cell fate on the composite surface. The positive effect of MC material on osteoblastic cell differentiation was widely reported [11, 47, 55]. However, the immunoregulatory effect of MC was less documented. Recently, Shi *et al*. [56] compared morphology of the macrophages grown on culture glass and pure MC, respectively. He observed by the actin cytoskeleton staining that the macrophages tended to have oval or spindle shape with protrusions on the surface of culture glass. However, a rounder and more conserved morphology of the macrophages was presented when adhering to pure MC.

**Figure 8:**
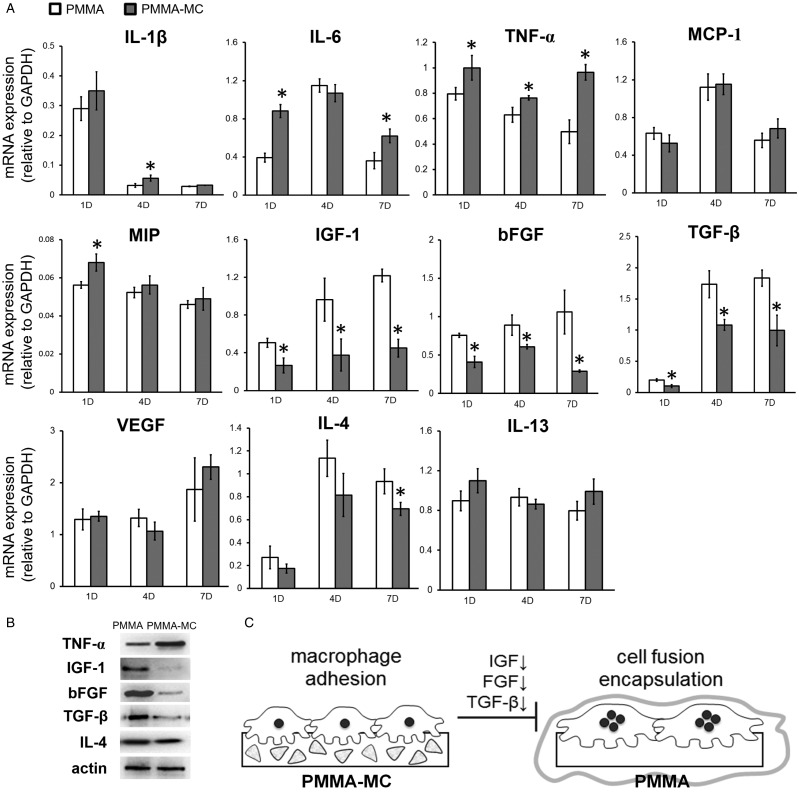
(**A**) qRT-PCR Analysis of the gene expression levels of pro-inflammatory cytokines, chemokines, fibroblast-stimulating growth factors and macrophage fusion related genes in macrophages cocultured with PMMA or PMMA-MC materials at days 1, 4 and 7, *P < 0.05 denotes for significant difference as compared to the PMMA group. (**B**) Western blotting confirmation of the differentially expressed genes. (**C**) Schematic illustration shows the influence of MC particles on fibrous encapsulation of PMMA

It is well accepted in implant design that a target for reduction of fibrous capsule thickness would be inhibition of macrophage fusion and synthesis of molecules with effect on fibroblasts [54, 57]. Bank *et al*. [58] discovered that capsules were thicker coincided with increased expression of fibroblast-stimulating growth factors. Critical factors secreted by macrophages during implantation include pro-inflammatory cytokines (Interleukins and TNF family), chemokines (MCP, MIP) and fibroblast-stimulating growth factors (IGF, FGF, TGF, VEGF). Therefore, in the current study, gene expressions of these factors are examined at days 1, 4 and 7 (Fig. 8A). We found consistently lower gene expressions of IGF-1, bFGF and TGF-β in the macrophage cultured on PMMA-MC throughout the culture time, as compared to those cultured on unmodified PMMA. Western blotting analysis further confirmed a decrease at protein level of these gene expressions (Fig. 8B). These gene expressions are known to be positively correlated with formation of fibrous encapsulation that a high gene expression corresponds with a thicker capsule size [58]. Our results also revealed promotion of IL-6 and TNF-α gene expressions in the PMMA-MC group. These genes are reported to have osteogenic effects in various aspects, that is enhancing osteoclastogenesis, promoting osteoblast differentiation and migration of MSCs [59, 60]. Furthermore, we found a decreased IL-4 gene and protein expression in the PMMA-MC group at the end of the study. IL-4 plays a critical role in macrophage fusion [61]. It is known to inhibit the RANKL-induced osteoclast differentiation while at the same time promoting macrophage fusion to form multinucleated giant cells [62]. A lower IL-4 cytokine level in the PMMA-MC cocultured macrophage medium is consistant with less fusion events in our result. There were two phenotypes in macrophage polarization, M1 and M2. IL-4 is mainly produced by M2 macrophage and less secreted by M1 macrophage [63]. It was reported that extrafibrillarly-MC would promote M1 polarization of macrophage, which might be the reason of a lower IL-4 and a higher IL-6 expression in the MC modified group [64, 65]. IL-13 is another identified cytokine which may also promote macrophage fusion [61]. However, there was no statistical difference in IL-13 expression between PMMA-MC and PMMA group in the current study. Together, our findings demonstrated that the reduced fibrous capsule formation on the surface of modified bone cement group is possibly related to the MC induced decrease in macrophage fusion, proliferation and a prominent down-regulation effect on fibrotic genes (Fig. 8C).

## Conclusions

In the present study, an injectable MC incorporated PMMA-based composite have been successfully prepared as a material for percutaneous cement discoplasty surgery to treat lumbar DDD. The introduction of MC could significantly improve hydrophilicity and dynamic mechanical performance of PMMA as a bone substitute. *In vivo* study with a goat L3–L4 degenerative disc model confirmed that the osteointegration of the MC-modified PMMA was greatly enhanced in terms of circumferential contact length between bone and cement. While heavy fibrous tissue encapsulation was identified around the pure PMMA cement, lesser was found in the bone covered PMMA-MC group. In further *in vitro* study, we found that the addition of MC would hinder the proliferation and fusion of the macrophages. At both gene and protein levels, expressions of several fibroblast-stimulating growth factors were significantly reduced in the PMMA-MC coculture. Together with the good injectability, the prominent osteoconductive ability of the MC-modified PMMA material makes it an attractive candidate for invasive PCD surgery. 

## Funding

This work was supported by National Natural Science Foundation of China (grant no. 81971755), Sichuan Science and Technology Innovation Team of China (2019JDTD0008), Young Elite Scientist Sponsorship Program by CAST (2019QNRC001), Fundamental Research Funds for the Central Universities, “111” Project of China (B16033), Key Research and Development Project of Heibei Province (182777172), Provincial Key Technology Support Program of Sichuan (grant no. 2015SZ0027), and Graduate Student’s Research and Innovation Fund of Sichuan University (Grant No. 2018YJSY067).

*Conflict of interest statement*. None declared.
